# The Brain-Derived Neurotrophic Factor Val66Met Polymorphism Can Protect Against Cognitive Impairment in Multiple Sclerosis

**DOI:** 10.3389/fneur.2021.645220

**Published:** 2021-03-16

**Authors:** Emilio Portaccio, Angelo Bellinvia, Elio Prestipino, Benedetta Nacmias, Silvia Bagnoli, Lorenzo Razzolini, Luisa Pastò, Claudia Niccolai, Benedetta Goretti, Mattia Fonderico, Giovanni Bosco Zimatore, Nunzia Alessandra Losignore, Sandro Sorbi, Maria Pia Amato

**Affiliations:** ^1^SOD Riabilitazione Neurologica, AOU Careggi, Florence, Italy; ^2^IRCCS Fondazione Don Carlo Gnocchi, Florence, Italy; ^3^NEUROFARBA Department, University of Florence, Florence, Italy; ^4^Ente Ecclestiastico Ospedale Generale Regionale “F. Miulli”, Acquaviva delle Fonti, Italy

**Keywords:** multiple sclerosis, cognitive impairment, disability, brain derived neurotrophic factor, polymorphism

## Abstract

**Introduction:** Brain-derived neurotrophic factor (BDNF) is a member of the neurotrophin family, involved in neuronal survival and synaptic plasticity. The BDNF Val66Met polymorphism is known to reduce BDNF expression and secretion; its role in multiple sclerosis (MS) is poorly investigated.

**Objectives and Methods:** In this multicenter, retrospective study, we assessed the role of BDNF Val66Met polymorphism on cognitive and motor disability in MS patients consecutively referred to the University of Florence and the Hospital of Barletta. All patients underwent a genetic analysis for the presence of Val66Met polymorphism and a comprehensive neuropsychological examination on the Rao's Brief Repeatable Battery and the Stroop Color Word Test. Possible predictors of the Expanded Disability Status Scale (EDSS) score and number of failed neuropsychological tests were assessed through linear multivariable regression models.

**Results:** Ninety-eight patients were recruited. Patients with the BDNF Val66Met polymorphism (35.7%) were more frequently males (*p* = 0.020), more disabled (*p* = 0.026) and, marginally, older (*p* = 0.064). In the multivariable analysis, BDNF Val66Met polymorphism was associated with a better cognitive performance (*B* = −1.1 ± 0.5, *p* = 0.027). Higher EDSS score was associated with a progressive disease course (*B* = 3.4, *p* < 0.001) and, marginally, with the presence of the BDNF Val66Met polymorphism (*B* = 0.56, *p* = 0.066).

**Discussion:** Our results preliminarily suggest a protective role of BDNF Val66Met polymorphism against cognitive impairment in MS patients, possibly related to a detrimental effect of increased BDNF concentration in a neuroinflammatory environment.

## Introduction

Multiple sclerosis (MS) is an inflammatory and neurodegenerative disorder of the central nervous system (CNS) that affects mainly patients between 20 and 40 years of age. It is the second cause of neurological disability in the young adult population, after trauma (1). Cognitive impairment (CI) is widely acknowledged as a core feature of MS, affecting up to 70% of the patients, with a significant functional impact in everyday activities (2). In adult patients, information processing speed, attention, working and episodic memory, executive functions, and visuospatial abilities are the cognitive domains most commonly impaired, with relative sparing of language and general intelligence (2, 3). CI in MS has been linked to different risk factors (4): among genetic factors, the role of brain-derived neurotrophic factor (BDNF) polymorphisms is receiving growing attention.

BDNF is a member of the neurotrophin family, which also includes nerve growth factor and neurotrophins 3 and 4. BDNF is secreted from dendrites to axons and from axons to dendrites, in autocrine loops, and across long distances through neural circuits (5, 6). BDNF is involved in different processes within the brain, such as plasticity, neuronal survival, formation of new synapses, dendritic branching, and modulation of excitatory and inhibitory neurotransmitter profiles (7).

The BDNF single-nucleotide polymorphism *rs6265* (also named Val66Met) determines the substitution of valine with methionine at codon 66 of the BDNF pro-protein (8). Its presence leads to interference with BDNF intracellular trafficking and secretion, as it has been demonstrated in *in vitro* studies (8, 9). The presence of the abovementioned polymorphism also results in an 18–30% reduction in BDNF secretion (9). The Val66Met polymorphism has been reported to be a risk factor for neurodegenerative disorders (such as Alzheimer's disease) in the adult age (10). In addition, it has been associated with CI in otherwise healthy individuals, particularly with involvement of episodic and working memory, which require neuroplasticity, and hence abundant expression of BDNF in related brain areas (8, 11–14).

In neuroinflammation, the role of BDNF is entangled with the effects of factors involved in the innate and adaptive immune response in neurodegenerative and autoimmune disorders (15), inducing BDNF expression and secretion by immune cells. The role of BDNF in neuroinflammatory disorders, and especially in MS, has been poorly investigated so far, with conflicting results (16). In the first study assessing the role of BDNF Val66Met polymorphism on magnetic resonance imaging (MRI) parameters in a group of MS patients, Met carriers showed a higher risk of developing gray matter (GM) atrophy (17). Conversely, other subsequent studies showed that Met carriers had a higher preservation of brain volume (18) and global and regional GM volumes (19, 20). On the other hand, a large-scale Norwegian study found no role of the BDNF Val66Met polymorphism on clinical and neuropsychological variables (21).

With this background, the aim of the present cross-sectional multicenter study was to assess the influence of Val66Met polymorphism on both cognitive and motor disability in a sample of MS patients.

## Materials and Methods

### Subjects

Patients with MS consecutively referred to the MS Centres at the University of Florence and the Hospital of Barletta between 2014 and 2019 were screened for inclusion. Inclusion criteria were as follows: diagnosis of MS according to the 2010 McDonald's Diagnostic Criteria (22), relapsing-remitting (RR), or progressive (either primary progressive, PP, or secondary progressive, SP) disease course; age >18 years; and no history of intellectual disability, psychosis, or dementia. Exclusion criteria were corticosteroid treatment in the 30 days before inclusion and inability or refusal to perform the blood sampling required for the study purposes. The study was approved by the local Ethic Committees, and written informed consent was obtained by all the subjects.

### Clinical and Neuropsychological Examination

In each center, demographic and clinical data were prospectively collected every 6 months and in occasion of relapses and stored in an electronic database (23). For this cross-sectional analysis, at the time of assessment and blood sampling, the following demographic and clinical data were collected by a qualified neurologist: age, sex, education, age at disease onset, disease course, ongoing treatments, relapses in the last year, and disability level as measured on the Expanded Disability Status Scale (EDSS) (24). A well-trained psychologist administered the Brief Repeatable Battery of Neuropsychological Tests (BRB) (25) and the Stroop Color Word Test (SCWT). The BRB assesses the cognitive domains most frequently impaired in MS and incorporates tests of verbal memory [Selective Reminding Test (SRT)], visuo-spatial memory [10/36 Spatial Recall Test (SPART)], complex attention and information processing speed [Paced Auditory Serial Addition Test (PASAT) and Symbol Digit Modalities Test (SDMT)], and verbal fluency [Word List Generation (WLG)]. The SCWT (26) assesses complex attention and aspects of executive functioning such as the ability to inhibit cognitive interference. Failure of a test was defined as a score below the 5th or above the 95th percentile (1.65 SD), as appropriate, on the basis of Italian normative values after adjustment for age, sex, and education (27). Premorbid intelligent quotient (IQ) was estimated through the Italian version of the National Adult Reading Test (NART)—the “Test di Intelligenza Breve” (28). Finally, fatigue and depression were assessed through the Fatigue Severity Scale (29) and the Montgomery and Asberg Depression Rating Scale (30), respectively.

### Genetic Analysis

A blood sample for genetic analysis of the BDNF Val66Met polymorphism was obtained from each patient. The presence of the *rs6265* polymorphism was analyzed by first extracting the DNA from peripheral blood samples, using a standardized, automated method (QIAcube, QIAGEN). After DNA extraction, a high-resolution melting analysis (HRMA) method was used to analyze the presence of *rs6265* polymorphism, using the following primers: 5′-ACTCTGGAGAGCGTGAATGG-3′ and 5′-ACTACTGAGCATCACCCTGGA-3′ for the polymerase chain reaction (PCR) to amplify the subjects' DNA.

### Statistical Analysis

Demographic and clinical characteristics were described as frequency (percentage) and mean ± standard deviation (SD). Group comparisons were assessed through the Pearson's chi-square, Student *t*, and Mann–Whitney *U* tests when appropriate. Possible predictors of the number of failed neuropsychological tests were assessed through a backward stepwise linear regression model, including as covariates BDNF genotype, sex, age, education, disease duration, disease course, number of relapses in the year before inclusion, EDSS, treatment, and premorbid IQ. *P* < 0.05 were considered as significant. Likewise, possible predictors of EDSS score were assessed through a backward stepwise linear regression model, including as covariates BDNF genotype, sex, age, disease duration, disease course, number of relapses in the year before inclusion, and treatment.

## Results

Ninety-eight patients were included in the analysis, 80 (81.6%) with a RR, 12 (12.3%) with a SP, and six (6.1%) with a PP course. SP and PP patients were analyzed as a whole group, named chronic progressive (CP) MS. The genetic analysis identified 35 (35.7%) patients with the BDNF *rs6265* (Val/Met) polymorphism. The main demographical and clinical characteristics of the whole sample and of the two groups (Val/Met and Val/Val) are depicted in Table 1. In the univariate analysis, patients with the BDNF *rs6265* polymorphism were more frequently males (48.6 vs. 24.4%, *p* = 0.020, chi-squared test), more disabled (median EDSS score 3, IQR: 2–5.5 vs. 2, IQR 1.5–4.5, *p* = 0.026, Mann–Whitney *U* test), and, marginally, older (46.7 ± 10.7 vs. 42.3 ± 10.8 years, *p* = 0.064, *t*-test for independent samples) than Val/Val patients. Eighty-eight (89.8%) patients were treated with disease-modifying therapies (DMTs) (two azathioprine; 17 interferon in its various formulations; nine glatiramer acetate; 39 dimethyl fumarate; three teriflunomide; 10 natalizumab; one methotrexate; one cyclophosphamide; six fingolimod) (31).

**Table 1:** Characteristics of the study sample.

	**Whole sample (*n* = 98)**	**BDNF Val/Met (*n* = 35)**	**BDNF Val/Val (*n* = 63)**	***p***
Females, *n* (%)^a^	65 (66.3%)	18 (51.4%)	47 (74.6%)	**0.020**
Age, mean years (SD)^b^	43.9 (10.9)	46.7 (10.7)	42.3 (10.8)	0.064
Education, mean years (SD)^b^	12.1 (3.96)	11.5 (3.8)	12.5 (4.0)	0.213
IQ, median (IQR)^c^	108.8 (101.6–111.9)	108.2 (98.0–114.0)	109.3 (103.6–111.4)	0.885
Disease course				0.392
RR, *n* (%)^a^	80 (81.6)	27 (77.1)	53 (84.1)	
CP, *n* (%)^a^	18 (18.4)	8 (22.9)	10 (15.9)	
Age at onset, mean years (SD)^b^	35.3 (10.4)	36.9 (10.8)	34.4 (10.2)	0.262
Disease duration, mean years (SD)^b^	8.6 (7.4)	9.9 (8.5)	7.8 (6.7)	0.225
EDSS score, median (IQR)^c^	2 (1.5–5.5)	3 (2–5.5)	2 (1.5–4.5)	**0.026**
No of relapses in the past year, mean (SD)^b^	0.4 (0.6)	0.26 (0.5)	0.4 (0.7)	0.244
Treated with DMTs, *n* (%)^a^	88 (89.8%)	32 (91.4%)	56 (88.9%)	0.691
FSS, median (IQR)^c^^*^	5.0 (3.6–6.0)	4.6 (3.7–5.6)	5.2 (2.9–6.1)	0.449
MADRS, median (IQR)^c^	6.0 (4.0–9.0)	6.0 (4.0–9.0)	6.0 (4.0–8.0)	0.676
No of failed tests, mean (SD)^b^	1.9 (2.3)	1.5 (1.8)	2.13 (2.5)	0.051
SRT-LTS score, median (IQR)^c^	38.2 (28.2–45.4)	38.2 (32.2–46.3)	36.2 (25.4–43.4)	0.184
SRT-CLTR score, median (IQR)^c^	27.4 (19.9–39.1)	27.4 (22.1–39.1)	27.4 (19.1–38.1)	0.784
SRT-D score, median (IQR)^c^	7.7 (5.0–9.3)	7.9 (5.3–9.5)	7.5 (4.9–9.3)	0.580
SPART score, median (IQR)^c^	18.7 (15.4–23.7)	19.2 (15.9–23.8)	17.8 (13.9–21.9)	0.252
SPARTD score, median (IQR)^c^	6.3 (4.9–7.9)	6.9 (5.2–8.9)	6.3 (4.9–7.3)	**0.025**
SDMT score, median (IQR)^c^	48.4 (42.2–57.3)	49.2 (42.5–57.6)	47.4 (41.5–57.2)	0.477
WLG score, median (IQR)^c^	23.0 (18.0–26.7)	21.1 (16.9–26.9)	23.1 (18.9–26.9)	0.333
ST score, median (IQR)^c^	53.5 (41.4–63.2)	51.1 (34.6–61.7)	54.3 (47.8–63.7)	0.221
Pasat2 score, median (IQR)^c^	25.5 (8.3–34.4)	29.3 (16.9–34.2)	203.3 (4.9–34.8)	0.104
Pasat3 score, median (IQR)^c^	40.2 (27.7–49.0)	38.4 (28.9–49.6)	40.5 (23.9–46.5)	0.417

* *available in 68 subjects*;

a *Chi-squared*;

b *t-test for independent samples*;

c *Mann–Whitney U test*.

*IQ, intelligence quotient; RR, relapsing-remitting; CP, chronic progressive; EDSS, Expanded Disability Status Scale; DMTs, disease-modifying treatments; FSS, Fatigue Severity Scale; MADRS, Montgomery and Asberg Depression Rating Scale; SRT, Selective Reminding Test; SRT-LTS, Selective Reminding Test–Long Term Storage; SRT-CLTR, Selective Reminding Test–Consistent Long Term Retrieval; SRT-D, Selective Reminding Test–Delayed; SPART, Spatial Recall Test; SPART-D, Spatial Recall Test–Delayed; SDMT, Symbol Digit Modalities Test; WLG, Word List Generation; ST, Stroop Test; PASAT-2, Paced Auditory Serial Addition Test−2 seconds; PASAT-3, Paced Auditory Serial Addition Test−3 seconds; SD, standard deviation. The bold values are the statistically significant ones (p < 0.05)*.

As for cognitive assessment, at the univariate analysis, Val/Met patients demonstrated a trend toward a lower number of failed neuropsychological tests as opposed to Val/Val patients (1.5 ± 1.8 vs. 2.1 ± 2.5, *p* = 0.051, *t*-test for independent samples). Moreover, BDNF Val/Met polymorphism patients had higher mean adjusted score on the SPART-D (median 6.9, IQR: 5.2–8.9 vs. 6.3, IQR: 4.9–7.3, *p* = 0.025, Mann–Whitney *U* test). There were no differences in the other adjusted scores obtained in the remaining neuropsychological tests. The same was true when comparing the MADRS and FSS scores obtained by Met carriers and Val/Val homozygotes.

In the multivariable analysis, the presence of the BDNF *rs6265* Val/Met polymorphism (*B* = −1.1 ± 0.5, *p* = 0.027) and, marginally, a higher IQ (*B* = −0.6 ± 0.03, *p* = 0.068) were associated with a lower number of failed cognitive tests. On the other hand, higher EDSS score was associated with a higher mean number of failed neuropsychological tests (*B* = 0.385 ± 0.128, *p* = 0.003). The *R*-square for the model was 21.1%, with an adjusted *R*-square for the overall model of 18.4%, a medium size effect according to Cohen (32) (Table 2).

**Table 2:** Predictors of number of failed neuropsychological tests based on a linear regression model.

	***β***	***p***
EDSS	0.385	**0.003**
IQ	−0.6	0.068
BDNF *rs6265* (Val/Met) polymorphism	−1.1	**0.027**

*Adjusted R-square for the model: 0.184. EDSS, Expanded Disability Status Scale; IQ, intelligence quotient; BDNF, brain-derived neurotrophic factor. Covariates that were not retained in the final model are as follows: disease course, sex, age, disease duration, treatment with disease-modifying therapies, education, and number of relapses in the last year. The bold values are the statistically significant ones (p < 0.05)*.

As for disability, a higher EDSS score was associated with a CP course (*B* = 3.4, *p* < 0.001), while there was a trend toward an association with Val/Met polymorphism (*B* = 0.56, *p* = 0.066). Other variables included in the model were sex, age, disease duration, mean number of relapses in the last year, and treatment. The *R*-square for the model was 48.4%, with an adjusted R-square for the overall model of 47.3%, a large size effect according to Cohen (32) (Table 3).

**Table 3:** Predictors of EDSS score based on a linear regression model.

	***β***	***p***
CP course	3.382	** <0.001**
BDNF *rs6265 (Val/Met)* polymorphism	0.559	0.066

*Adjusted R-square for the model: 0.473. EDSS, Expanded Disability Status Scale; CP, chronic progressive; BDNF, brain-derived neurotrophic factor. Covariates that were not retained in the final model are as follows: sex, age, disease duration, treatment with disease-modifying therapies, and number of relapses in the last year. The bold values are the statistically significant ones (p < 0.05)*.

## Discussion

While BDNF has been consistently associated with better cognitive performances in healthy individuals and was found to be a protective factor against memory impairment in neurodegenerative disorders (such as Alzheimer disease) (10), its role in neuroinflammatory diseases is still poorly understood. In MS, previous studies on possible relationships between BDNF and both cognitive and motor disability have reported conflicting results (17–20). In our cross-sectional multicenter study, we assessed the role of the BDNF *rs6265* polymorphism on cognitive functions and disability among MS patients.

Carriers of Met allele showed an overall better cognitive performance, failing a lower number of neuropsychological tests. The strength of this association, which was marginal at the univariate analysis, significantly increased after adjustment for well-acknowledged demographic and clinical confounders of cognitive functioning in MS (in particular age and disability, which were unevenly distributed between the two groups) (33).

Our results are in line with another study exploring the role of BDNF Val/Met polymorphism in MS on MRI parameters and cognitive performances on the PASAT, a test of information processing speed and complex attention (19). Indeed, in that study, Met carriers had both higher GM volumes and better cognitive performances than Val/Val carriers. The same protective role of BDNF *rs6265* polymorphism against brain atrophy was highlighted in other subsequent studies (18, 20). Moreover, in a recent functional-MRI study, the BDNF Val/Met polymorphism was associated with increased functional connectivity between the hippocampus and posterior cingulate cortex in comparison with Val homozygosis during retrieval phase of an episodic memory task, while the opposite was true for healthy controls (34).

In general, conversely to what has been demonstrated in the general population and neurodegenerative diseases, findings from our and the abovementioned studies suggest a protective role of BDNF Val/Met polymorphism against cognitive decline and brain atrophy in MS patients. The impact of BDNF in MS could potentially be very different from that in healthy individuals and other pathological conditions, due to differences in the pathophysiological milieu in which BDNF exerts its effects. The neuroinflammatory environment of the MS lesions contains immune cells, such as infiltrating T-cells and macrophages, as well as activated astrocytes. These cells were found to express higher BDNF mRNA levels (35–37), contributing to increased BDNF secretion. In addition, BDNF could have a dual role in the setting of neuroinflammation, depending on its concentration. For instance, neurons populating the edges of active lesions and oligodendrocytes (and their precursors) have been found to express higher levels of two different BDNF receptors, the TrKB BDNF receptor (35) and the p75 neurotrophin receptor (NTR) (38), respectively. These two receptors have different affinities for BDNF and mediate different effects of this molecule. In particular, the high-affinity TrKB receptor (active at low BDNF concentrations) mediates the signaling cascade connected with neuronal survival, while the low-affinity p75 NTR (binding with BDNF at higher concentrations) is thought to mediate a pro-apoptotic role. Therefore, an increased production of BDNF in a neuroinflammatory milieu can have a detrimental effect, shifting the balance toward apoptosis, and neurodegeneration.

Furthermore, BDNF is known to facilitate glutamatergic synaptic transmission via mechanisms involving the N-methyl-D-aspartate receptors (39). This action has a crucial role for neuroplasticity and long-term potentiation, which are fundamental for learning and memory, and could account for the positive effect of BDNF in healthy population and neurodegenerative diseases (6, 12). On the other hand, in MS patients, LTP activation and glutamate excitotoxicity can cause oligodendrocytes and neuronal loss (40). Additionally, neuronal processes requiring the activity-dependent component of BDNF could be compromised by the constitutive presence of the immune cell-derived BDNF.

Against this background, it could be argued that the presence of abundant BDNF in an inflammatory environment could be detrimental for neuronal functions, promoting toxicity mechanisms that could enhance synaptic degeneration. Taken as a whole, these actions can hinder cognitive functioning, contributing to neuropsychological impairment.

In our study, beyond BDNF polymorphism, CI was associated with greater disability levels as measured on the EDSS. This finding is consistent with the existing literature, showing that age and disability levels are the main drivers of neuropsychological dysfunction in MS (33).

As for motor disability, while a potential negative effect of Val/Met polymorphism emerged in the univariate analysis, in the multivariable analysis, the only significant predictor of higher EDSS score was the CP course of the disease. The neutral role of the BDNF *rs6265* polymorphism on disability in MS was also evident in a large cross-sectional study conducted in Norway including 2,149 MS patients (21). The absence of a significant relationship between BDNF polymorphism and motor disability can be due, at least in part, to the differential expression of BDNF in the CNS. Indeed, greater expression of BDNF has been reported in brain regions involved in learning and memory, such as the hippocampal formation and the prefrontal cortex, where the anatomical effect of Val66Met polymorphism is most apparent (8, 11, 12). It must be noted that other regulating factors, which were not assessed in our study, such as epigenetic mechanisms and DNA methylation, can modulate the effects of BDNF polymorphism. In a recent study on 209 MS patients, while the presence of Val/Met polymorphism was not linked to disability accumulation, a lower BDNF gene DNA methylation, and therefore, higher gene expression and BDNF secretion, was associated to a higher risk of reaching EDSS 6.0 (41). Whether higher BDNF expression is directly responsible of disability worsening or represents an ineffective compensatory attempt needs to be clarified.

In interpreting the study findings, a few limitations should be considered. The sample size was relatively small. In the univariate analysis, Met carriers were more frequently males, more disabled, and, marginally, older than Val/Val homozygotes, reflecting a possible sampling bias. These differences can account, at least in part, for the marginal association between BDNF polymorphism and motor disability, which disappeared in the multivariable model. On the other hand, older age and greater disability in Met carriers are expected to increase the proportion of CI in this group: in this respect, as commented above, our findings seem to reinforce the hypothesis of a protective effect of BDNF polymorphism against CI in MS. Moreover, data on MRI evaluations are lacking, as well as measurement of actual levels of BDNF at the time of clinical and neuropsychological evaluations. Finally, since genetics has influence during the course of the disease, the cross-sectional design prevented the assessment of a possible longitudinal effect of BDNF polymorphism on study outcomes.

Despite these limitations, our results suggest a protective role of BDNF Val66Met polymorphism against CI in MS patients, possibly reflecting a detrimental effect of increased BDNF concentration in a neuroinflammatory environment. These preliminary findings indicate that BDNF and its polymorphism may represent a potential biomarker for susceptibility and severity of CI in MS, as well as a possible therapeutic target of pharmacological interventions for neuropsychological dysfunction. Further studies are needed to confirm our findings on larger populations, with longitudinal MRI and clinical evaluations.

## Data Availability Statement

The datasets presented in this study can be found in online repositories. The names of the repository and accession number can be found here: Harvard Dataverse, accession link: https://dataverse.harvard.edu/loginpage.xhtml?redirectPage=%2Fdataset.xhtml%3FpersistentId%3Ddoi%3A10.7910%2FDVN%2FOVA6P9. Anonymized data will be shared on reasonable request from a qualified investigator.

## Ethics Statement

The studies involving human participants were reviewed and approved by Local Ethic Committes. The patients/participants provided their written informed consent to participate in this study.

## Author Contributions

EPo, AB, EPr, SS, and MA developed the original concept of the study, developed the analysis plan, and did the manuscript writing. EPo, LR, LP, MF, and GZ contributed to clinical data acquisition. CN, BG, and NL contributed to data acquisition and performed the neuropsychological assessment. BN and SB developed and performed laboratory analyses. All authors reviewed and commented on drafts of the protocol and paper. All authors read and approved the final manuscript.

## Conflict of Interest

EPr received compensation for travel grants, participation in advisory board, and/or speaking activities from Biogen, Merck Serono, Sanofi, Teva, and Novartis and serves on the editorial board of Frontiers in Neurology. LP received research support from Novartis, Biogen, and speaker honoraria from Teva. LR received research support from Novartis. GZ received travel funds and speaker honoraria from Sanofi-Genzyme, Novartis, Teva, Biogen, Almirall, Roche, and Merck. MA served on scientific advisory boards for and has received speaker honoraria and research support from Biogen Idec, Merck Serono, Bayer Schering Pharma, and Sanofi Aventis and serves on the editorial board of BMC Neurology. The remaining authors declare that the research was conducted in the absence of any commercial or financial relationships that could be construed as a potential conflict of interest. The Handling Editor declared a past co-authorship with some of the authors EPo, LR, and MA.
